# A small scale study on the effects of oral administration of the β-glucan produced by *Aureobasidium pullulans* on milk quality and cytokine expressions of Holstein cows, and on bacterial flora in the intestines of Japanese black calves

**DOI:** 10.1186/1756-0500-5-189

**Published:** 2012-06-19

**Authors:** Hirofumi Uchiyama, Atsushi Iwai, Yukoh Asada, Daisuke Muramatsu, Shiho Aoki, Koji Kawata, Kisato Kusano, Koji Nagashima, Daisuke Yasokawa, Mitsuyasu Okabe, Tadaaki Miyazaki

**Affiliations:** 1 Aureo Science Co., Ltd., Hokudai Business Spring, North 21, West 12, Kita-ku, Sapporo, Hokkaido, 001-0021, Japan; 2 Aureo Co., Ltd, 54-1 Kazusakoito, Kimitsu, Chiba, 292-1149, Japan; 3 Hokkaido Food Processing Research Center, 589-4 Bunkyodai, Midorimachi, Ebetsu, Hokkaido, 069-0836, Japan; 4 Department of Probiotics Immunology, Institute for Genetic Medicine, Hokkaido University, Kita-15, Nishi-7, Kita-Ku, Sapporo, 060-0815, Japan

## Abstract

**Background:**

The β–(1 → 3),(1 → 6)-D-glucan extracellularly produced by *Aureobasidium pullulans* exhibits immunomodulatory activity, and is used for health supplements. To examine the effects of oral administration of the β–(1 → 3),(1 → 6)-D-glucan to domestic animals, a small scale study was conducted using Holstein cows and newborn Japanese Black calves.

**Findings:**

Holstein cows of which somatic cell count was less than 3 x 10^5^/ml were orally administered with or without the β-(1 → 3),(1 → 6)-D-glucan-enriched *A. pullulans* cultured fluid (AP-CF) for 3 months, and the properties of milk and serum cytokine expression were monitored. Somatic cell counts were not significantly changed by oral administration of AP-CF, whereas the concentration of solid non fat in the milk tended to increase in the AP-CF administered cows. The results of cytokine expression analysis in the serum using ELISA indicate that the expressions of tumor necrosis factor-α (TNF-α) and interleukin (IL)-6 in all cows which were orally administered with AP-CF became slightly lower than that of control cows after the two-month treatment. On the other hand, IL-8 expression tended to indicate a moderately higher level in all treated cows after the three-month administration of AP-CF in comparison with that of the control cows. Peripartum Japanese Black beef cows and their newborn calves were orally administered with AP-CF, and bacterial flora in the intestines of the calves were analyzed by T-RFLP (terminal restriction fragment length polymorphism). The results suggest that bacterial flora are tendentiously changed by oral administration of AP-CF.

**Conclusions:**

Our data indicated the possibility that oral administration of the β–(1 → 3),(1 → 6)-D- glucan produced by *A. pullulans* affects cytokine expressions in the serum of Holstein cows, and influences bacterial flora in the intestines of Japanese Black calves. The findings may be helpful for further study on the efficacies of oral administration of β-(1 → 3),(1 → 6)-D-glucans on domestic animals.

## Findings

### Background

β-(1 → 3)-D-glucans produced by mushrooms, fungi and yeast are known to exhibit immunomodulating activity [1], and some beneficial effects of the β-(1 → 3)-D-glucans for cancer [2-4] and allergy [4,5] have been reported. A black yeast, *Aureobasidium pullulans* extracellularly produces β-(1 → 3)-D-glucan under a certain condition [6]. *A. pullulans* produces a β-(1 → 3)-D-glucan in a viscous water-soluble form, and in its structural feature, the β-(1 → 3)-D-glucan is known to be highly branched with β-(1 → 6)-glycosidic bonds [7,8]. The β-(1 → 3),(1 → 6)-D-glucan-containing *A. pullulans* cultured fluid is permitted as a food additive in many countries, and is used in health supplements. The β-(1 → 3),(1 → 6)-D-glucan produced by *A. pullulans* also exhibits immunomodulating activity and beneficial effects for various diseases just as the β-(1 → 3)-D-glucans derived from other organisms do [9-12]. The beneficial effect of oral administration of the β-(1 → 3),(1 → 6)-D-glucan produced by *A. pullulans* on health is believed not only to apply to humans, but also to animals. Thus, the β-(1 → 3),(1 → 6)-D-glucan-containing food supplements are also provided for pets. However, the effects of oral administration of the β-(1 → 3),(1 → 6)-D-glucan to domestic animals have not been well evaluated.

In this study, Holstein cows and Japanese Black calves were used to investigate the effect of oral administration of the β-(1 → 3),(1 → 6)-D-glucan produced by *A. pullulans* on domestic animals. The effects of this on the quality of milk, on cytokine expression profiles and on bacterial flora in the intestine were evaluated.

### Methods

#### Cattle

In total, 15 Holstein cows, 8 Japanese Black peripartum beef cows, and their 8 new born calves in collaborating farms were used in this study. The β-(1 → 3),(1 → 6)-D-glucan produced by *A. pullulans* was administered as cattle feed. The β-(1 → 3),(1 → 6)-D-glucan has been approved as a cattle feed in Japan. All experiments in this study were conducted as part of daily management of cattle in the farms, and were performed after obtaining approval from the owners of the cattle. Hence, all procedures which involved contact with cattle, such as blood sampling and feces sampling, were performed by veterinarians as medical activities for daily management of the animals’ health. This study did not require ethical approval in Japan. However, all experiments were conducted in consideration of the animals’ welfare.

#### Preparation of *A. Pullulans*-cultured fluid (AP-CF) and partial purified β-(1 → 3),(1 → 6)-D-glucan

*A. pullulans* was grown at 24.5°C for 10 days, in a medium containing rice bran and sucrose, as a nitrogen and a carbohydrate source respectively. After the cultured medium was heated at 90°C for 30 min, the heat-sterilized cultured medium was used as AP-CF in this study.

The purified β-(1 → 3),(1 → 6)-D-glucan was prepared from the water soluble fraction of *A. pllulans* cultured fluid by ultrafiltration (a cut-off molecular weight of 200,000; Advantec, Tokyo, Japan). This fraction is assumed to be minimally containing other compounds, such as mono- and oligo-saccharides, nucleotides, and soluble proteins.

#### Monitoring milk quality

The somatic cell counts and the concentrations of solid non fat in the milk were measured by the Tokachi Federation of Agricultural Cooperatives, Tokachi, Hokkaido, Japan (to evaluate the effects of orally administered AP-CF), and the Livestock Improvement Association of Japan, Tokyo, Japan (to evaluate the effects of orally administered purified β-(1 → 3),(1 → 6)-D-glucan).

#### ELISA

Blood samples were collected using vacuum tubes from the Holstein cows by doctors of veterinary medicine. After the serum was separated by centrifugation, the serum expression levels of interleukin (IL)-6, interleukin-8 (also called CXCL-8) and TNF-α (tumor necrosis factor-α) were analyzed using commercially available ELISA kits (Cusabio Biotech, Wuhan, China) in accordance with the manufacturer's protocols.

#### T-RFLP (terminal restriction fragment length polymorphism) analysis and construction of the dendrogram

T-RFLP analysis was performed as previously described [13,14]. The T-RFLP results were subjected to the cluster analysis using UPGMA (unweighted pair-group method with arithmetic mean). The dendrogram was constructed using a web-based program (DendroUPGMA; http://genomes.urv.es/UPGMA/) [15].

### Results and discussion

#### The effects of oral administration of the β-(1 → 3),(1 → 6)-D-glucan on somatic cell count and solid non fat in the milk of Holstein cows

**Figure 1:**
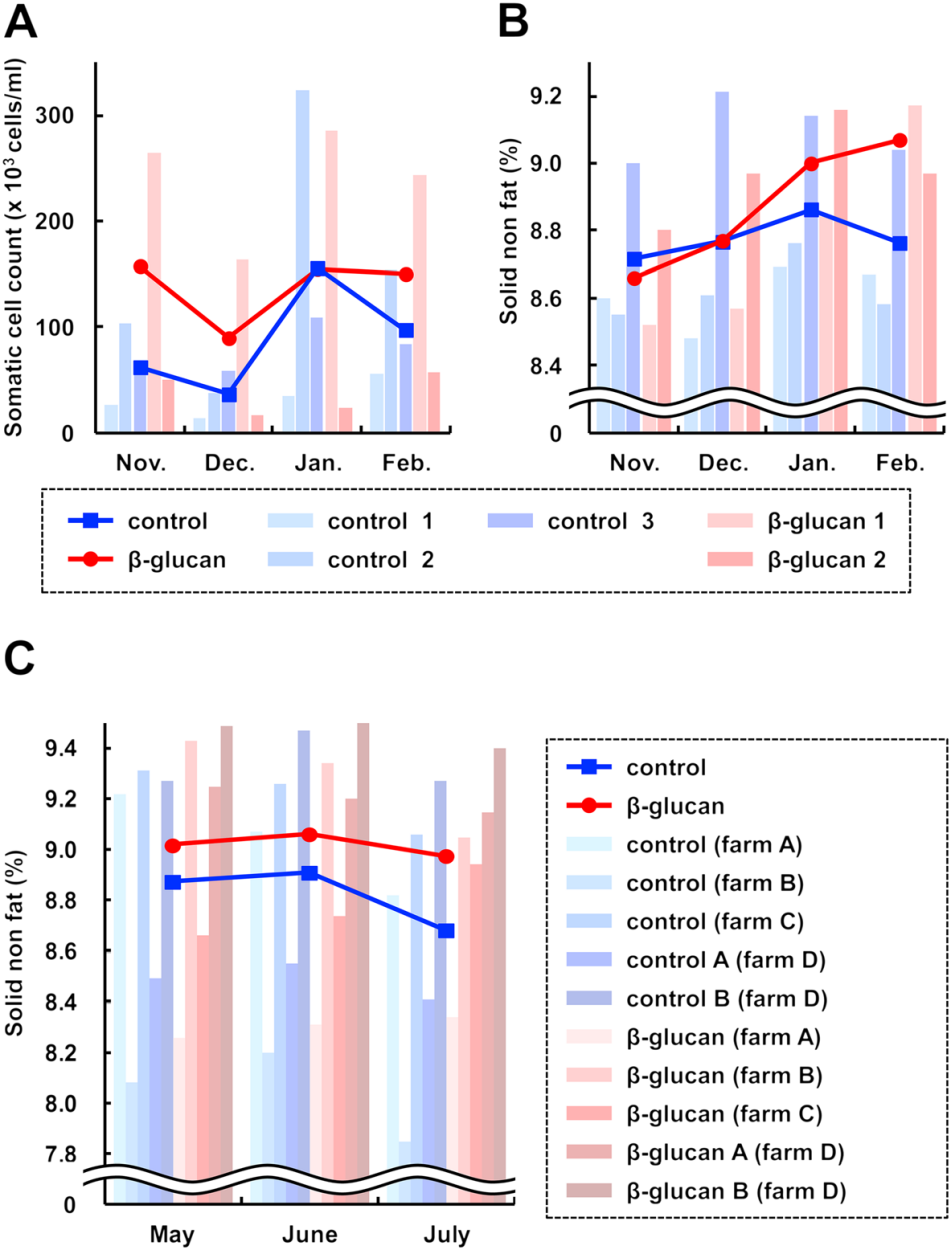
**The effects of oral administration of the β-(1 → 3),(1 → 6)-D-glucan on somatic cell count and solid non fat in the milk of Holstein cows. (A, B)** Milk-producing Holstein cows were orally administered the β–(1 → 3),(1 → 6)-D-glucan-enriched *A. pullulans* cultured fluid (AP-CF) for three months, and then the somatic cell count **(A)** and solid non fat **(B)** in the milk were monitored every month. Milk produced from untreated cows was used as a control. The diagnoses of individual milk are indicated with a bar graph, and the line graph represents the mean. **(C)** The result of the same experiment with panel B conducted at other farms. A partially purified β-(1 → 3),(1 → 6)-D-glucan was used for this experiment.

Initially, we examined the effect of oral administration of the β-(1 → 3),(1 → 6)-D-glucan on the quality of milk produced by Holstein cows. The lactating Holstein cows of which somatic cell count was less than 3 x 10^5^/ml were used for this experiment. The Holstein cows were orally administered with (n = 2) or without (n = 3) the β-(1 → 3),(1 → 6)-D-glucan-enriched *A. pullulans*-cultured fluid (AP-CF; 300 ml/cow, 0.42 g/dl β-(1 → 3),(1 → 6)-D-glucan) once a day for three months, and the somatic cell counts and solid non fat in the milk were monitored. The results show that the initial difference of somatic cell counts between the administered cows and the control cows was not significantly changed throughout the experiment (Figure 1A). On the other hand, after the three-month administration of AP-CF, the mean concentration of solid non fat in the milk from the administered cows was higher than that of the control cows, and the difference of the mean between the administered cows and the control cows increased (Figure 1B).

Similar results were obtained by an experiment using purified β-(1 → 3),(1 → 6)-D-glucan in other farms (Figure 1C). The Holstein cows were orally administered with (n = 5) or without (n = 5) the partially purified β-(1 → 3),(1 → 6)-D-glucan (80 ml/cow, 0.42 g/dl β-(1 → 3),(1 → 6)-D-glucan) for two months, and the concentrations of solid non fat in the milk were monitored. As shown in Figure 1C, the results show that the mean concentration of solid non fat in the milk from control cows decreased in June, while the purified β-(1 → 3),(1 → 6)-D-glucan administered cows had no decrease in concentration.

The data shown in Figure 1B and 1C indicate that oral administration of the β-(1 → 3),(1 → 6)-D-glucan does not seem to be effective for the cows producing milk which has a relatively high concentration of solid non fat (≥9.0%). However, the concentration of solid non fat showed a tendency to increase with oral administration of the β-(1 → 3),(1 → 6)-D-glucan to cows producing milk containing a relatively low concentration of solid non fat (<9.0%). The results may indicate the possibility that oral administration of the β-(1 → 3),(1 → 6)-D-glucan exhibits efficacy to improve the solid non fat of Holstein cow milk.

#### The evaluation of the effect on cytokine expressions in the serum by oral administration of the β-(1 → 3),(1 → 6)-D-glucan

**Figure 2:**
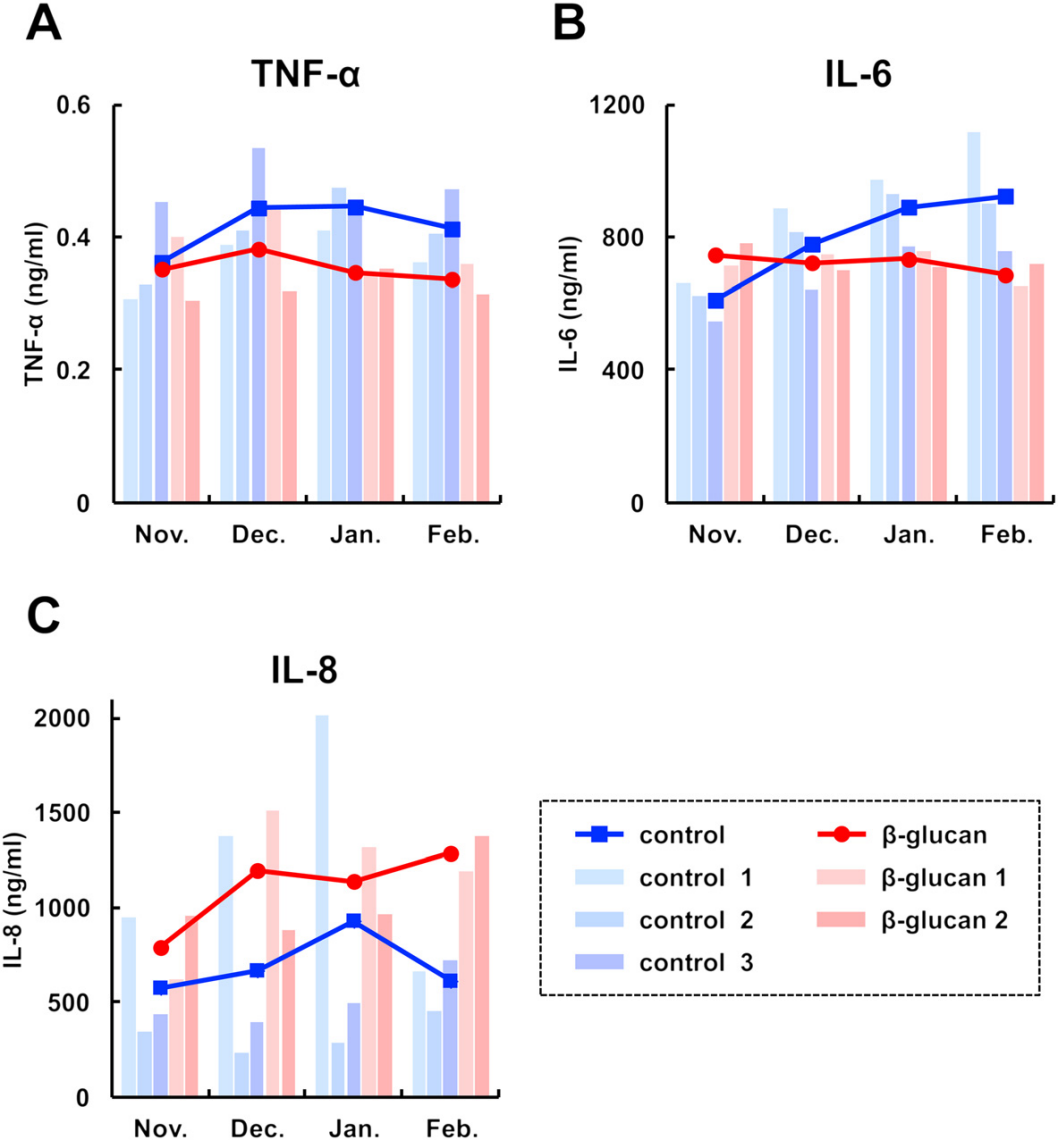
**The effects of oral administration of the β-(1 → 3),(1 → 6)-D-glucan on cytokine expressions in the serum of Holstein cows.** Serum was sampled from the Holstein cows used in Figure 1A and B, and then the expressions of tumor necrosis factor-α (TNF-α; **A**), interleukin (IL)-6 **(B)**, and IL-8 **(C)** in the serum were quantified by ELISA. The bar graphs indicate the quantification value of each serum, and the line graphs show the mean.

To evaluate the effect of oral administration of the β-(1 → 3),(1 → 6)-D-glucan on cytokine expressions, blood samples were collected from the same Holestein cows used in Figure 1A and 1B, and the serum expression levels of interleukin (IL)-6, interleukin-8 (also called CXCL-8) and TNF-α (tumor necrosis factor-α) were analyzed by ELISA. Overall, the results show that the expressions of these inflammatory cytokines were not strongly affected by oral administration of AP-CF. However, after a two-month administration, TNF-α (Figure 2A) and IL-6 (Figure 2B) expressions in all cows which were orally administered with AP-CF became slightly lower than that of the control cows. On the other hand, the expression of IL-8 in all of the AP-CF administered cows was greater after a three-month administration than with that of the control cows (Figure 2C). It is known that TNF-α is involved in the induction of IL-8 expression [16-18]. However, the increment of IL-8 expression in AP-CF administered cows seems to be independent of the TNF-α expression (Figure 2A and 2C). Although the mechanism remains unclear, the results may indicate the possibility that basal IL-8 expression in the serum is increased after long-term oral administration of the β-(1 → 3),(1 → 6)-D-glucan.

IL-8 is also known to be a chemokine belonging to the CXC chemokine family, and is responsible for migration of neutrophils [19]. An abnormal accumulation of neutrophils is frequently found in various inflammatory diseases, and this accumulation causes tissue damage through the excess production of superoxide from neutrophils [20]. However, neutrophils play a pivotal role in the early immune response in the elimination of extracellular pathogens. Therefore, the activation of neutrophils in an appropriate level is thought to be important in order to protect from infectious diseases. Although the function of IL-8 expression induced by oral administration of the β-(1 → 3),(1 → 6)-D-glucan to Holstein cows remains unclear, there might be a possibility that the increment of IL-8 expression exhibits protective activity against an infection of extracellular pathogens through the activation of neutrophils in an appropriate condition.

#### The effect of oral administration of the β-(1 → 3),(1 → 6)-D-glucan on bacterial flora in the intestines of Japanese black calves

**Figure 3:**
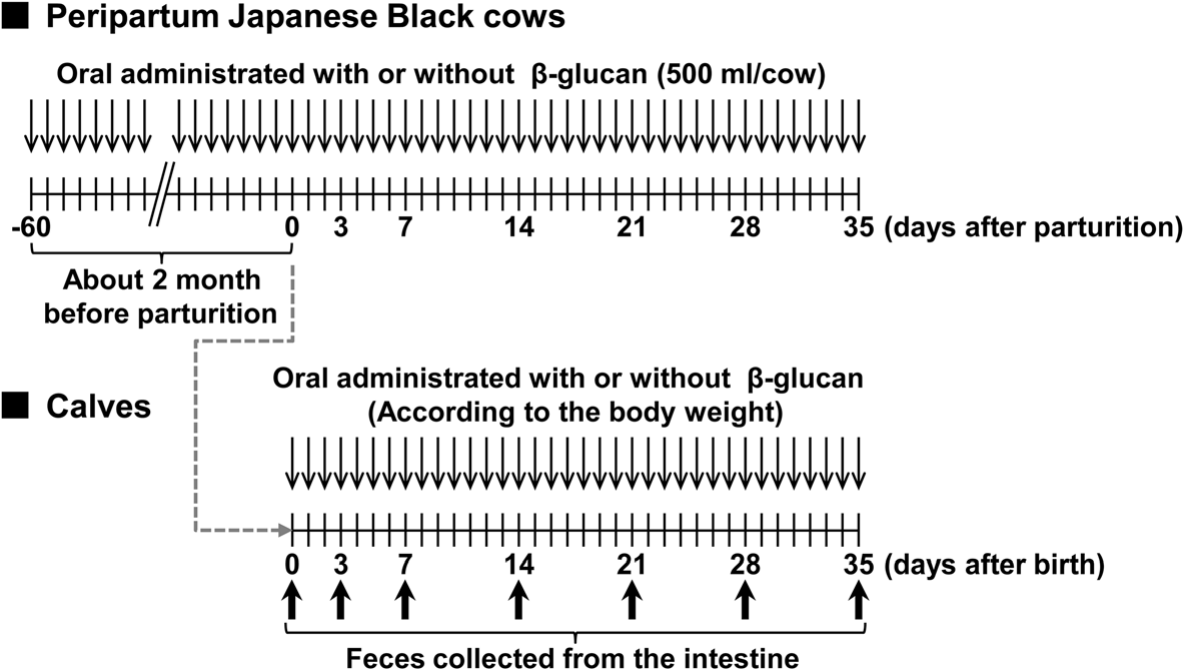
**The experimental procedure to estimate the effects of the β-(1 → 3),(1 → 6)-D-glucan on bacterial flora in the intestines of Japanese Black calves.** The amounts of AP-CF orally administered to the calves are indicated in Table 1.

**Figure 4:**
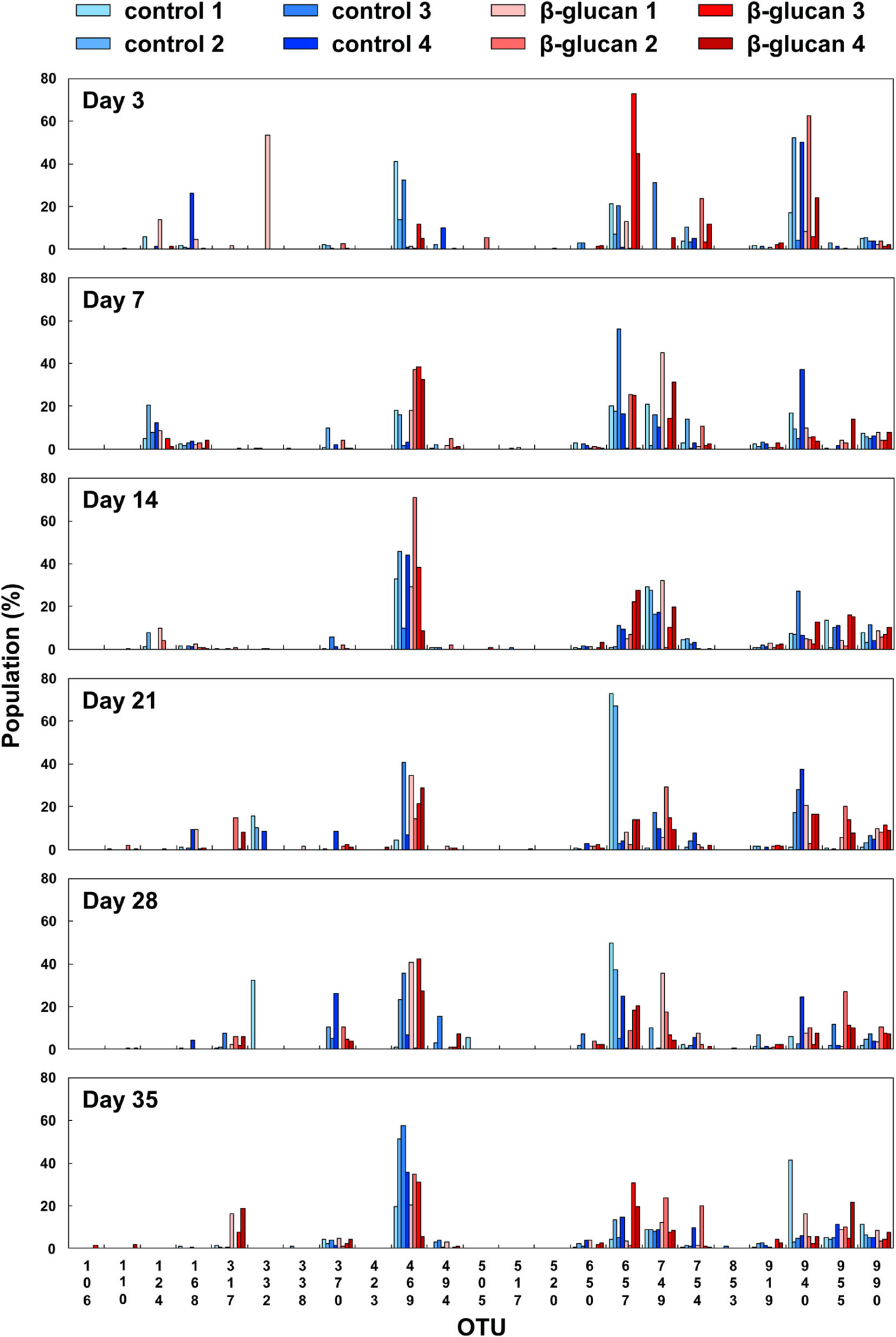
**T-RFLP analysis of bacterial flora in the intestines of Japanese Black calves.** The correspondence of operational taxonomic units (OTUs) to phylogenetic bacterial groups is listed in Table 2.

β-(1 → 3),(1 → 6)-D-glucans are known to be dietary fiber, and basically not digestible for mammals. Therefore, there is a possibility that oral administration of the β-(1 → 3),(1 → 6)-D-glucan affects bacterial flora in the intestines of cattle. Thus, to investigate the effects of oral administration of the β-(1 → 3),(1 → 6)-D-glucan on bacterial flora in the intestine, T-RFLP (terminal restriction fragment length polymorphism) analysis of the feces was performed [13, 14]. This experiment was conducted with Japanese Black peripartum beef cows and the experimental procedure to estimate the effects of the β-(1 → 3),(1 → 6)-D-glucan on bacterial flora in the intestine is summarized in Figure 3. The prepartum Japanese Black cows were orally administered with (n = 4) or without (n = 4) AP-CF (300 ml/cow, 0.42 g/dl β-(1 → 3),(1 → 6)-D-glucan) once a day beginning 60 days before the parturition. Oral administration was continued throughout the experiment. After birth, the calves (n = 4 for each group) were orally administered with AP-CF once a day for 35 days. As shown in Table 1, the amount of AP-CF used for oral administration was adjusted depending on the body weight of calf. The feces of calves were collected at 3, 7, 14, 21, 28 and 35 days after birth, and then the bacterial flora in the intestines of the cows were analyzed by T-RFLP. Correspondence of operational taxonomic units (OTUs) to phylogenetic bacterial groups is listed in Table 2. As shown in Figure 4, although there was great diversity of bacterial flora between individual calves, some tendencies were found after the 35-day administration of AP-CF. For instance, the population of the genus *Prevotella* (317-bp OTU) tended to increase in the calves administered with AP-CF in comparison with that of non-administered calves.

**Table 1:** **The amount of orally administrated the β-(1** → **3),(1** → **6)-D-glucan-enriched*A. pullulans*cultured fluid (AP-CF) to the calves**

**Body weight**	**Amount of AP-CF**
**(kg)**	**(ml)**
**30**	**15**
**35**	**30**
**40**	**45**
**45**	**60**
**50~**	**75**

**Table 2:** Correspondence of OTUs to phylogenetic bacterial groups

**OTU**	**Phylogenetic bacterial groups**
**106**	***Clostridium*subcluster XIVa**
**110**	***Clostridium*subcluster IX, Megamonas**
**124**	***Bifidobacterium***
**168**	***Clostridium*subcluster IV**
**317**	***Prevotella***
**332**	***Lactobacillales***
**338**	***Clostridium*subcluster XI**
**370**	***Clostridium*subcluster IV**
**424**	***Clostridium*subcluster XVIII**
**469**	***Bacteroides***
**494**	***Clostridium*subcluster XIVa**
**505**	***Clostridium*subcluster XIVa**
**517**	***Clostridium*subcluster XIVa**
**520**	***Lactobacillales***
**650**	***Clostridium*subcluster XVIII**
**657**	***Lactobacillales***
**749**	***Clostridium*subcluster IV**
**754**	***Clostridium*subcluster XIVa**
**853**	***Bacteroides***
**919**	***Clostridium*subcluster XI, XIVa**
**940**	***Clostridium*subcluster XIVa, *Enterobacteriales***
**955**	***Clostridium*subcluster XIVa**
**990**	***Clostridium*subcluster XIVa**

**Figure 5:**
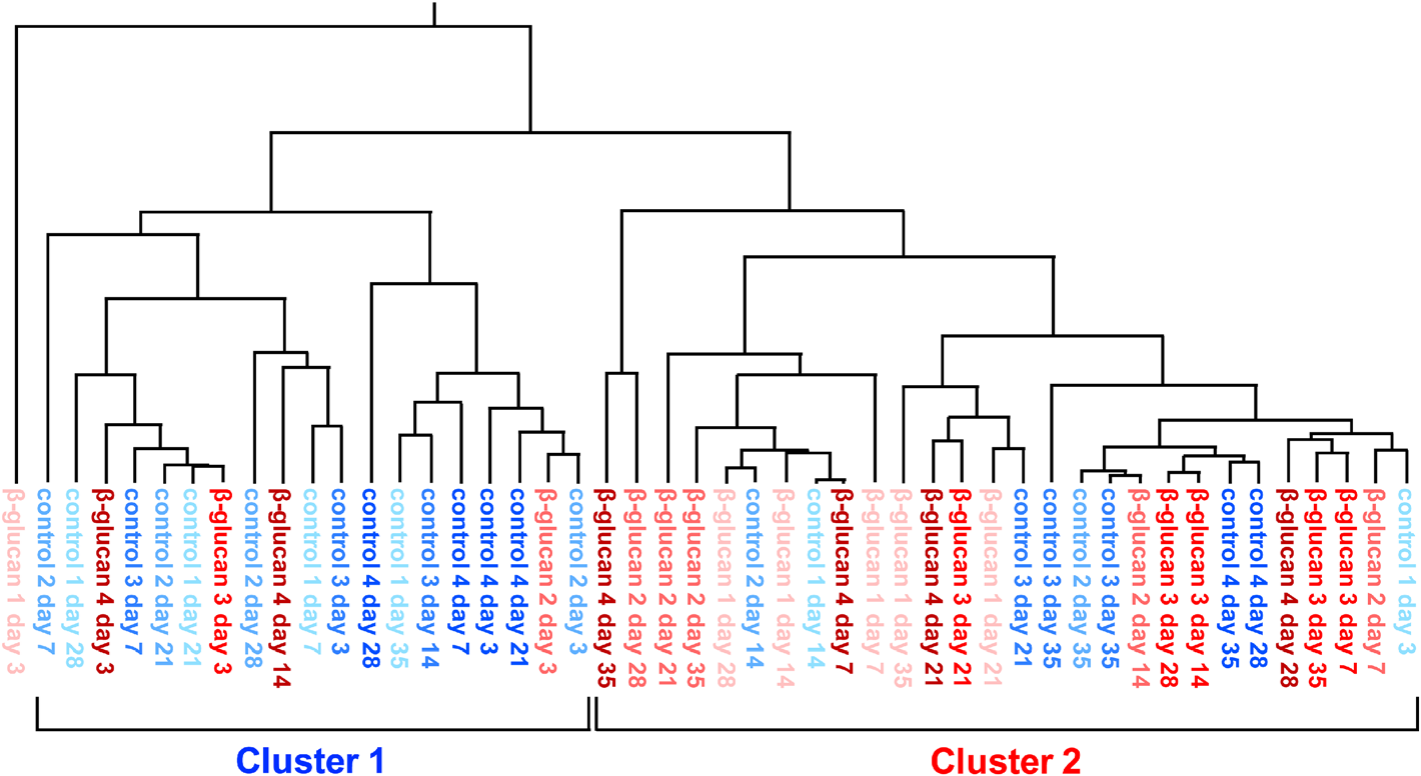
**The cluster analysis of bacterial flora in the intestines of the calves to estimate the effects of oral administration of the β-(1 → 3),(1 → 6)-D-glucan.** The results of the cluster analysis are shown by a dendrogram. The unweighted pair-group method with arithmetic mean (UPGMA) was used for the construction of the dendrogram.

To clarify the effect of oral administration of the β-(1 → 3),(1 → 6)-D-glucan on bacterial flora, the cluster analysis of the T-RFLP results was performed using UPGMA (unweighted pair-group method with arithmetic mean). The results show that the T-RFLP results were separated into two clusters, and most of the cows administered with AP-CF belong to cluster 2 whereas most of the control cows belong to cluster 1 (Figure 5). This may suggest that oral administration of the β-(1 → 3),(1 → 6)-D-glucan tends to affect bacterial flora of the intestine.

In this study, we examined the effects of oral administration of the β-(1 → 3),(1 → 6)-D-glucan produced by *A. pullulans* to cattle using Holstein cows and Japanese Black calves, and its possible effects on solid non fat in milk, on expressions of cytokines, and on bacterial flora in intestine were shown. The differences of concentration of solid non fat in the milk (Figure 1B and 1C) and expression of cytokines in the serum (Figure 2) between the β-(1 → 3),(1 → 6)-D-glucan administered cows and the control cows were found after a two or three-month administration. This may suggest that the effects of oral administration of the β-(1 → 3),(1 → 6)-D-glucan on cattle do not immediately appear. The influence of oral administration of the β-(1 → 3),(1 → 6)-D-glucan on bacterial flora might explain the reason. Although the effect of the change of bacterial flora in the intestine by oral administration of the β-(1 → 3),(1 → 6)-D-glucan on health has not been clarified, the influence of bacterial flora is thought to be an important phenomenon to evaluate the effects of the β-(1 → 3),(1 → 6)-D-glucan.

Basically, the β-(1 → 3),(1 → 6)-D-glucan is not digested by enzymes secreted in mammalians’ gastrointestinal tract. Thus, the β-(1 → 3),(1 → 6)-D-glucan is thought to work as a soluble dietary fiber in humans when taken as a food. On the other hand, in cattle, the orally administered β-(1 → 3),(1 → 6)-D-glucan would not only work as a soluble dietary fiber, it would have other effects. In ruminants including cattle, commensal microorganisms in the rumen are important in helping ruminant animals to gain nutrients from their food. This digestion mechanism for cellulose metabolism is thought to be important to understand the effects of oral administration of the β-(1 → 3),(1 → 6)-D-glucan to cattle. It is known that some fungi, yeasts, and bacteria have β-(1 → 3)-D-glucanases which are able to digest the β-(1 → 3),(1 → 6)-D-glucan. Therefore, the orally administered β-(1 → 3),(1 → 6)-D-glucan may affect the growth of certain bacterial strains which are able to use the β-(1 → 3),(1 → 6)-D-glucan as a nutrient, and this may influence the formation of bacterial flora in the intestine. In addition, the enhancement of β-(1 → 3)-D-glucanases activity may affect the production of oligosaccharides and glycoproteins from foods in the rumen. Thus, in cattle, the orally administered β-(1 → 3),(1 → 6)-D-glucan is thought to exhibit the effects through more complex mechanisms in comparison with those in humans.

Although our data shown in this study are suggestive, we believe that our data are helpful for further study on the effects of oral administration of β-(1 → 3),(1 → 6)-D-glucans on domestic animals. More large-scale and long-term studies are required to establish the efficacy of oral administration of the β-(1 → 3),(1 → 6)-D-glucan to maintain the health of domestic animals.

## Competing interests

H. Uchiyama, A. Iwai, D. Muramatsu and K. Kawata are employees of Aureo Science Co., Ltd., and Y. Asada, K. Kusano and M. Okabe are employees of Aureo Co., Ltd.

## Authors’ contributions

AI - Study design, literature search, data analysis, manuscript writing, editing and submission of the manuscript, HU, YA, DM, SA, KK, KK, KN, DY, MO and TM participated in Study design, data analysis, manuscript writing and editing. All the authors read and approved the final manuscript.

## References

[B1] Chan GC Chan WK Sze DM The effects of beta-glucan on human immune and cancer cells J Hematol Oncol 2009 2 25 10.1186/1756-8722-2-25 19515245 PMC2704234

[B2] Zhou LD Zhang QH Zhang Y Liu J Cao YM The shiitake mushroom-derived immuno-stimulant lentinan protects against murine malaria blood-stage infection by evoking adaptive immune-responses Int Immunopharmacol 2009 9 4 455 462 10.1016/j.intimp.2009.01.010 19189863

[B3] Liu J Gunn L Hansen R Yan J Yeast-derived beta-glucan in combination with anti-tumor monoclonal antibody therapy in cancer Recent Pat Anticancer Drug Discov 2009 4 2 101 109 10.2174/157489209788452858 19519533

[B4] Hetland G Johnson E Lyberg T Kvalheim G The Mushroom Agaricus blazei Murill Elicits Medicinal Effects on Tumor, Infection, Allergy, and Inflammation through Its Modulation of Innate Immunity and Amelioration of Th1/Th2 Imbalance and Inflammation Adv Pharmacol Sci 2011 157015 10.1155/2011/157015 PMC3168293 21912538

[B5] Sugiyama A Hata S Suzuki K Yoshida E Nakano R Mitra S Arashida R Asayama Y Yabuta Y Takeuchi T Oral administration of paramylon, a beta-1,3-D-glucan isolated from Euglena gracilis Z inhibits development of atopic dermatitis-like skin lesions in NC/Nga mice J Vet Med Sci 72 6 755 763 20160419 10.1292/jvms.09-0526

[B6] Hamada N Deguchi K Ohmoto T Sakai K Ohe T Yoshizumi H Ascorbic acid stimulation of production of a highly branched, beta-1,3-glucan by Aureobasidium pullulans K-1–oxalic acid, a metabolite of ascorbic acid as the stimulating substance Biosci Biotechnol Biochem 2000 64 9 1801 1806 10.1271/bbb.64.1801 11055380

[B7] Navarini L Bella J Flaibani A Gilli R Rizza V Structural characterization and solution properties of an acidic branched (1– > 3)-beta-D-glucan from Aureobasidium pullulans Int J Biol Macromol 1996 19 3 157 163 10.1016/0141-8130(96)01121-X 8910055

[B8] Tada R Tanioka A Iwasawa H Hatashima K Shoji Y Ishibashi K Adachi Y Yamazaki M Tsubaki K Ohno N Structural characterisation and biological activities of a unique type beta-D-glucan obtained from Aureobasidium pullulans Glycoconj J 2008 25 9 851 861 10.1007/s10719-008-9147-3 18587644

[B9] Kataoka-Shirasugi N Ikuta J Kuroshima A Misaki A Antitumor activities and immunochemical properties of the cell-wall polysaccharides from Aureobasidium pullulans Biosci Biotechnol Biochem 1994 58 12 2145 2151 10.1271/bbb.58.2145 7765709

[B10] Kimura Y Sumiyoshi M Suzuki T Sakanaka M Antitumor and antimetastatic activity of a novel water-soluble low molecular weight beta-1, 3-D-glucan (branch beta-1,6) isolated from Aureobasidium pullulans 1A1 strain black yeast Anticancer Res 2006 26 6B 4131 4141 17201124

[B11] Kimura Y Sumiyoshi M Suzuki T Suzuki T Sakanaka M Inhibitory effects of water-soluble low-molecular-weight beta-(1,3-1,6) d-glucan purified from Aureobasidium pullulans GM-NH-1A1 strain on food allergic reactions in mice Int Immunopharmacol 2007 7 7 963 972 10.1016/j.intimp.2007.03.003 17499199

[B12] Yatawara L Wickramasinghe S Nagataki M Takamoto M Nomura H Ikeue Y Watanabe Y Agatsuma T Aureobasidium-derived soluble branched (1,3-1,6) beta-glucan (Sophy beta-glucan) enhances natural killer activity in Leishmania amazonensis-infected mice Korean J Parasitol 2009 47 4 345 351 10.3347/kjp.2009.47.4.345 19967081 PMC2788712

[B13] Nagashima K Hisada T Sato M Mochizuki J Application of new primer-enzyme combinations to terminal restriction fragment length polymorphism profiling of bacterial populations in human feces Appl Environ Microbiol 2003 69 2 1251 1262 10.1128/AEM.69.2.1251-1262.2003 12571054 PMC143637

[B14] Nagashima K Mochizuki J Hisada T Suzuki S Shimomura K Fecal Microbiota and Improved Utility of Terminal Restriction Fragment Length Polymorphism Profiling Bioscience and Microflora 2006 25 3 99 107

[B15] Garcia-Vallve S Palau J Romeu A Horizontal gene transfer in glycosyl hydrolases inferred from codon usage in Escherichia coli and Bacillus subtilis Mol Biol Evol 1999 16 9 1125 1134 10.1093/oxfordjournals.molbev.a026203 10486968

[B16] Larsen CG Anderson AO Oppenheim JJ Matsushima K Production of interleukin-8 by human dermal fibroblasts and keratinocytes in response to interleukin-1 or tumour necrosis factor Immunology 1989 68 1 31 36 2478449 PMC1385501

[B17] Sheron N Williams R IL-8 as a circulating cytokine: induction by recombinant tumour necrosis factor-alpha Clin Exp Immunol 1992 89 1 100 103 1628417 10.1111/j.1365-2249.1992.tb06885.x PMC1554411

[B18] Aloisi F Care A Borsellino G Gallo P Rosa S Bassani A Cabibbo A Testa U Levi G Peschle C Production of hemolymphopoietic cytokines (IL-6, IL-8, colony-stimulating factors) by normal human astrocytes in response to IL-1 beta and tumor necrosis factor-alpha J Immunol 1992 149 7 2358 2366 1382099

[B19] Zlotnik A Yoshie O Chemokines: a new classification system and their role in immunity Immunity 2000 12 2 121 127 10.1016/S1074-7613(00)80165-X 10714678

[B20] Eyles JL Roberts AW Metcalf D Wicks IP Granulocyte colony-stimulating factor and neutrophils–forgotten mediators of inflammatory disease Nat Clin Pract Rheumatol 2006 2 9 500 510 10.1038/ncprheum0291 16951705

